# Tumor Microenvironment‐Based Risk Stratification of Oropharyngeal Squamous Cell Carcinoma

**DOI:** 10.1002/hed.27945

**Published:** 2024-09-28

**Authors:** Alhadi Almangush, Lauri Jouhi, Caj Haglund, Jaana Hagström, Antti A. Mäkitie, Ilmo Leivo

**Affiliations:** ^1^ Department of Pathology University of Helsinki Helsinki Finland; ^2^ Research Program in Systems Oncology, Faculty of Medicine University of Helsinki Helsinki Finland; ^3^ Department of Pathology University of Turku Turku Finland; ^4^ Faculty of Dentistry Misurata University Misurata Libya; ^5^ Department of Otorhinolaryngology – Head and Neck Surgery University of Helsinki and Helsinki University Hospital Helsinki Finland; ^6^ Research Programs Unit, Translational Cancer Medicine University of Helsinki Helsinki Finland; ^7^ Department of Surgery University of Helsinki and Helsinki University Hospital Helsinki Finland; ^8^ Department of Oral Pathology and Radiology, University of Turku Turku University Hospital Turku Finland; ^9^ Division of Ear, Nose and Throat Diseases, Department of Clinical Sciences, Intervention and Technology Karolinska Institutet and Karolinska University Hospital Stockholm Sweden; ^10^ Institute of Biomedicine, Pathology University of Turku Turku Finland; ^11^ Turku University Central Hospital Turku Finland

**Keywords:** oropharyngeal squamous cell carcinoma, prognosis, tumor microenvironment, tumor‐infiltrating lymphocytes, tumor‐stroma ratio

## Abstract

**Background:**

Evaluation of the prognostic impact of tumor microenvironment (TME) has received attention in recent years. We introduce a TME‐based risk stratification for oropharyngeal squamous cell carcinoma (OPSCC).

**Material and Methods:**

A total of 182 patients treated for OPSCC at the Helsinki University Hospital were included. TME‐based risk stratification was designed combining tumor‐stroma ratio and stromal tumor‐infiltrating lymphocytes assessed in hematoxylin and eosin‐stained sections.

**Results:**

In multivariable analysis, TME‐based risk stratification associated with poor disease‐free survival with a hazard ratio (HR) of 2.68 (95% CI 1.11–6.48, *p* = 0.029). In addition, the proposed risk stratification was associated with poor disease‐specific survival (HR 2.687, 95% CI 1.28–5.66, *p* = 0.009) and poor overall survival (HR 2.21, 95% CI 1.23–3.99, *p* = 0.008).

**Conclusion:**

Our TME‐based risk stratification provides a powerful prognostic tool that can be used in daily treatment planning of OPSCC together with tumor‐related prognostic markers.

## 1 Introduction

Oropharyngeal squamous cell carcinoma (OPSCC) is one of the most commonly occurring malignancies in the head and neck region. There is an increasing incidence of human papillomavirus‐associated (HPV+) OPSCC tumors [1, 2]. In general, HPV+ OPSCC is associated with a better prognosis than HPV− OPSCC, however many HPV+ cases present also with a poor survival [3]. HPV+ OPSCC patients who are at high risk of recurrence (about 15% of the cases), would require more intensive therapy, but their identification is challenging [2]. Therefore, there is a need for additional prognostic markers beyond the HPV status to predict the clinical behavior of OPSCC.

The tumor stroma as part of the tumor microenvironment (TME) has a prominent role in the progression of many cancer types including those of various subsites of head and neck region, as widely reported recently [4]. Tumor stroma consists of fibroblasts, myofibroblasts, endothelial cells, and immune cells. The stromal tissue serves as a supporting framework where the tumor cells are embedded [5].

Assessment of the quantity of stromal compartment has been recently introduced in the form of tumor‐stroma ratio (TSR) using hematoxylin and eosin (HE)‐stained sections and it has shown to have a powerful prognostic value [6, 7, 8, 9]. Moreover, immune cells may infiltrate tumor tissue, and their organization within the TME is tightly connected with the clinical behavior of many solid cancers [10]. Of note, the combination of TSR and immune status in TME has also been studied in some tumor types including head and neck cancer [11, 12].

Up to date, there are no TME‐related classifiers included in the prognostication of OPSCC. TME‐based stratification could improve accuracy in the assessment of the clinical behavior of OPSCC. Therefore, the aim of this study was to introduce a TME‐based risk stratification for the prognostication of OPSCC.

## 2 Material and Methods

All cases treated for OPSCC at the Helsinki University Hospital (Helsinki, Finland) in the period between January 2000 and December 2009 were included in this study. This research project was approved by the Research Ethics Committee of the Helsinki University Hospital. The following patients were excluded: those with palliative treatment intent (*n* = 44), with treatment for previous head and neck cancer (*n* = 11), with concurrent head and neck cancer (*n* = 5), other histology than squamous cell carcinoma (*n* = 18), and those in whom a sufficient tumor‐host interface was not available (*n* = 71). A total of 182 cases of OPSCC were eligible for this study. We used both tumor resection specimens and representative incisional diagnostic specimens. All unrepresentative samples were excluded. Both p16 immunohistochemistry and Ventana Inform DNA in situ hybridization assay were performed on tissue samples and used to determine HPV status based on the algorithm described by Smeets et al. [13].

**FIGURE 1:**
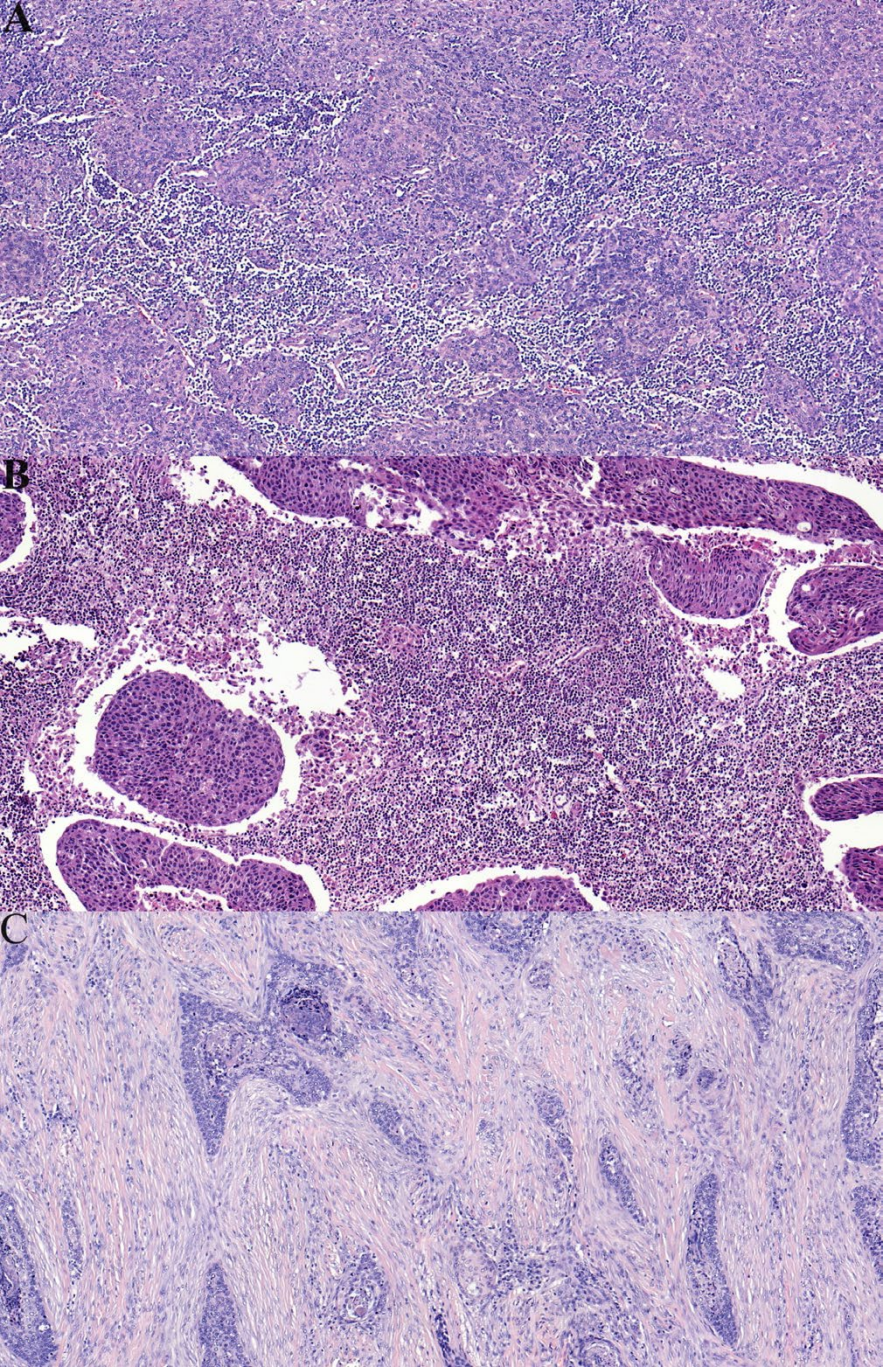
Tumor microenvironment‐based risk stratification of oropharyngeal squamous cell carcinoma. (A) Category I: Tumor with low stroma (< 50%) and high infiltration of TILs (≥ 30%). (B) Example of category II: High stroma (≥ 50%) and high infiltration of TILs (≥ 30%). (C) Category III: High stroma (≥ 50%) and low infiltration of TILs (< 30%). [Color figure can be viewed at wileyonlinelibrary.com]

All representative HE‐stained cancer specimens were assessed. TME‐based risk stratification was designed based on the abundance of tumor stroma and the stromal TILs (Figure 1). TSR and stromal TILs were combined as follows: category I in which the stromal component was less than 50% and TILs more than or equal to 30%, and category III in which the stromal component was more than or equal to 50% and TILs less than 30%. All other tumors were assigned to category II.

The evaluation of TSR and TILs was performed by two observers (AA and IL) as described in the recent guidelines [14, 15, 16, 17]. In brief, the assessment of TSR started with scanning of the whole slide with ×5 objective to select the area with the highest amount of tumor‐associated stroma, and then with ×10 objective to assess the amount of tumor‐associated stroma in a chosen microscopic field with cancer cells present in all four sides [14, 15]. In any heterogenous tumor with areas of both high and low amounts of tumor‐associated stroma, the stroma‐high area was considered decisive for scoring the case, as recommended in the guidelines [14, 15]. For the assessment of TILs, the whole tumor section was evaluated at low magnification using ×5 or ×10 objective, and then at higher magnification using ×20 objective. The percentage of stromal area occupied by TILs was assessed for scoring. To obtain an average score of TILs this assessment was carried out in multiple stromal areas. Stromal areas not adjacent to the tumor, tonsillar lymphatic tissue and areas of necrosis were excluded.

### 2.1 Statistical Method

All statistical analyses was conducted using IBM SPSS Statistics (version 27). The prognostic impact of the TME‐based risk stratification was assessed with univariable and multivariable analyses. Hazard radio (HR) with 95% confidence interval (CI) was reported for each variable. Cross‐tabulation was used to analyze the relationship between TME‐based stratification and the clinicopathologic characteristics. Kaplan–Meier analyses were conducted for disease‐free survival, disease‐specific survival, and overall survival.

## 3 Results

A total of 140 (76.9%) males and 42 (23.1%) females were included in the study. The median follow‐up time was 4.48 years (range 3.51–5.00 years). The clinicopathologic features of the cases and their relationship with the TME‐based risk stratification are summarized in Table 1. The TME‐based stratification had a total of 81 (44.5%) tumors categorized in the TME‐based category I, 39 (21.4%) in category II, and 62 (34.1%) in category III.

**TABLE 1:** Relationship between tumor microenvironment‐based risk stratification and clinicopathologic features in cases of oropharyngeal squamous cell carcinoma (*n* = 182).

Variable	Total	Stromal category I	Stromal category II	Stromal category III	*p* of Chi‐square test
	Total, *N* = 182	Number (%)	Number (%)	Number (%)	
		81 (44.5%)	39 (21.4%)	62 (34.1%)	
Age					0.006
< 60 years	101	55 (54.5%)	15 (14.9%)	31 (30.7%)	
≥ 60 years	81	26 (32.1%)	24 (29.6%)	31 (38.3%)	
Gender					0.688
Male	140	61 (43.6%)	29 (20.7%)	50 (35.7%)	
Female	42	20 (47.6%)	10 (23.8%)	12 (28.6%)	
HPV status					0.252
Positive	91	46 (50.5%)	18 (19.8%)	27 (29.7%)	
Negative	91	35 (38.5%)	21 (23.0%)	35 (38.5%)	
Smoking					0.550
Never	20	9 (45.0%)	3 (15.0%)	8 (40.0%)	
Former	46	21 (45.7%)	13 (28.3%)	12 (26.1%)	
Current	85	33 (38.8%)	19 (22.4%)	33 (38.8%)	
Stage					0.262
Early (I–II)	27	10 (37.0%)	9 (33.3%)	8 (29.6%)	
Advanced (III–IV)	155	71 (45.8%)	30 (19.4%)	54 (34.8%)	
T stage					0.174
T1	35	22 (62.9%)	7 (20.0%)	6 (17.1%)	
T2	68	30 (44.1%)	15 (22.1%)	23 (33.8%)	
T3	40	15 (37.5%)	10 (25.0%)	15 (37.5%)	
T4	39	14 (35.9%)	7 (17.9%)	18 (46.2%)	
N stage					0.601
N0	35	13 (37.1%)	9 (25.7%)	13 (37.1%)	
N+	147	68 (46.3%)	30 (20.4%)	49 (33.3%)	
Grade					0.301
I	15	5 (33.3%)	6 (40.0%)	4 (26.7%)	
II	70	29 (41.4%)	13 (18.6%)	28 (40.0%)	
III	97	47 (48.5%)	20 (20.6%)	30 (30.9%)	
Treatment					0.157
Sx ± (C)RT	120	58 (48.3%)	21 (17.5%)	41 (34.2%)	
(C)RT ± Sx	62	23 (37.1%)	18 (29.0%)	21 (33.9%)	

Abbreviations: CRT, chemoradiotherapy; RT, radiotherapy; Sx, surgery.

There was a good inter‐observer agreement in the assessment of TILs (Kappa value = 0.78) and TSR (Kappa value = 0.752), which indicates a good reproducibility of the proposed TME‐based stratification. In the cross‐tabulation (Table 1), we noted a significant association was noted between the age of patients and the TME‐based stratification (*p* = 0.006). On the other hand, no significant association (*p* > 0.05) was found between the TME‐based risk stratification and other clinicopathologic factors including gender, HPV status, smoking history, TNM stage, histopathologic grade, and treatment.

**FIGURE 2:**
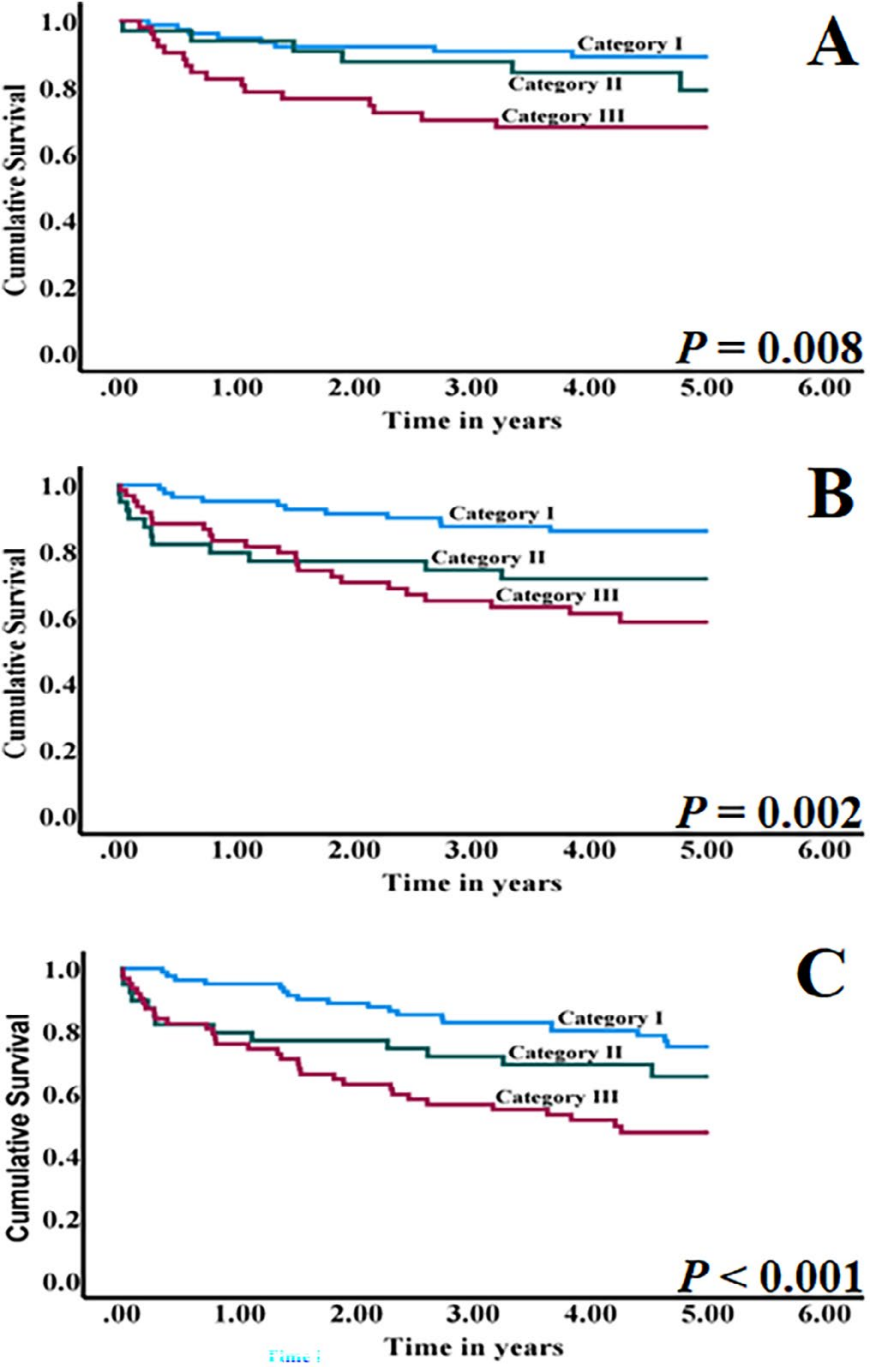
Kaplan–Meier survival curves for oropharyngeal squamous cell carcinoma cases categorized by tumor microenvironment‐based risk stratification. Cases with category III tumor microenvironment‐based stratification are associated with poor disease‐free survival (A), disease‐specific survival (B), and overall survival (C). [Color figure can be viewed at wileyonlinelibrary.com]

In survival analyses using a cutoff point of 30% for TILs (Table 2), category III of the TME‐based risk stratification was associated with significantly worse disease‐free survival with a HR of 3.52 (95% CI 1.50–8.22, *p* = 0.004) in univariable and multivariable analyses (HR 2.68, 95% CI 1.11–6.48, *p* = 0.029). Similarly, category III of TME‐based stratification was associated with significantly worse disease‐specific survival with a HR of 3.43 (95% CI 1.67–7.05, *p* < 0.001) in univariable and multivariable analyses (HR 2.687, 95% CI 1.28–5.66, *p* = 0.009). In addition, category III of the TME‐based risk stratification was associated with poor overall survival in univariable analysis with a HR of 2.83 (95% CI 1.60–4.99, *p* < 0.001) as well as in multivariable analysis (HR 2.21, 95% CI 1.23–3.99, *p* = 0.008). Our multivariable analyses included the routinely considered parameters of HPV‐status and tumor stage. The results indicate the independence of the proposed TME‐based risk stratification in predicting the prognosis of OPC. In addition, Kaplan–Meier survival curves (Figure 2A–C) indicated significantly worse prognosis for category III of TME‐based stratification in disease‐free survival (*p* = 0.008), disease‐specific survival (*p* = 0.002), and overall survival (*p* < 0.001).

**TABLE 2:** Disease‐free survival, disease‐specific survival, and overall survival analyses of the prognostic significance of tumor microenvironment (TME)‐based risk stratification and clinicopathologic parameters of 182 patients treated for oropharyngeal squamous cell carcinoma.

Parameter	Univariable analysis		
	Disease‐free survival	Disease‐specific survival	Overall survival
	HR (95% CI), *p*	HR (95% CI), *p*	HR (95% CI), *p*
Gender			
Male	Reference	Reference	Reference
Female	2.08 (0.80–5.39), *p* = 0.13	2.19 (0.99–4.88), *p* = 0.054	1.50 (0.85–2.64), *p* = 0.16
Smoking			
Never	Reference	Reference	Reference
Former	1.69 (0.47–6.15), *p* = 0.42	1.66 (0.46–6.05), *p* = 0.44	1.24 (0.48–3.21), *p* = 0.65
Current	1.89 (0.56–6.42), *p* = 0.31	3.29 (1.01–10.7), *p* = 0.048	2.36 (1.01–5.53), *p* = 0.048
T stage			
T1	Reference	Reference	Reference
T2	1.58 (0.51–4.89), *p* = 0.43	1.97 (0.74–5.27), *p* = 0.18	1.92 (0.84–4.43), *p* = 0.12
T3	2.34 (0.74–7.47), *p* = 0.15	1.79 (0.62–5.26), *p* = 0.28	2.44 (1.03–5.81), *p* = 0.044
T4	2.35 (0.69–8.04), *p* = 0.17	3.62 (1.31–9.96), *p* = 0.013	4.18 (1.79–9.76), *p* = 0.001
N stage			
N0–N1	Reference	Reference	Reference
N2–N3	1.55 (0.59–4.01), *p* = 0.37	2.09 (1.05–4.19), *p* = 0.037	1.49 (0.89–2.48), *p* = 0.129
HPV status			
Positive	Reference	Reference	Reference
Negative	2.59 (1.26–5.35), *p* = 0.010	2.51 (1.38–4.56), *p* = 0.003	2.46 (1.52–3.98), *p* < 0.001
Treatment			
Sx ± (C)RT	Reference	Reference	Reference
(C)RT ± Sx	1.24 (0.62–2.49), *p* = 0.551	1.01 (0.56–1.82), *p* = 0.98	1.13 (0.71–1.81), *p* = 0.604
TME‐based stratification			
Category I	Reference	Reference	Reference
Category II	1.79 (0.62–5.14), *p* = 0.383	2.39 (1.04–5.52), *p* = 0.041	1.65 (0.81–3.34), *p* = 0.165
Category III	3.52 (1.50–8.22), ***p*** = **0.004**	3.43 (1.67–7.05), ***p*** < **0.001**	2.83 (1.60–4.99), ***p*** < **0.001**

Parameter	Multivariable analysis		
	Disease‐free survival	Disease‐specific survival	Overall survival
	HR (95% CI), *p*	HR (95% CI), *p*	HR (95% CI), *p*
T stage			
T1	Reference	Reference	Reference
T2	1.77 (0.54–5.82), *p* = 0.35	2.22 (0.79–6.22), *p* = 0.13	2.21 (0.88–5.58), *p* = 0.09
T3	1.56 (0.46–5.29), *p* = 0.47	1.43 (0.48–4.25), *p* = 0.52	1.90 (0.73–4.95), *p* = 0.19
T4	1.95 (0.55–6.86), *p* = 0.30	2.76 (0.97–7.89), *p* = 0.06	3.72 (1.47–9.41), *p* = 0.006
HPV status			
Positive	Reference	Reference	Reference
Negative	2.56 (1.18–5.57), *p* = 0.02	2.98 (1.55–5.71), *p* = 0.001	2.59 (1.52–4.42), *p* < 0.001
TME‐based stratification			
Category I	Reference	Reference	Reference
Category II	1.94 (0.66–5.69), *p* = 0.225	2.696 (1.16–6.29), *p* = 0.022	1.74 (0.85–3.55), *p* = 0.127
Category III	2.68 (1.11–6.48), ***p*** = **0.029**	2.687 (1.28–5.66), ***p*** = **0.009**	2.21 (1.23–3.99), ***p*** = **0.008**

*Note*: Values in bold refers to the significant prognostic power of TME‐Based stratification.

When using a cutoff point of 20% for TILs in the TME‐based risk stratification, a significant prognostic power was observed for disease‐free survival (HR 2.80, 95% CI 1.78–6.66, *p* = 0.020). However, no significant prognostic power was reached for disease‐specific survival (HR 1.45, 95% CI 0.75–2.81, *p* = 0.271) or overall survival (HR 1.47, 95% CI 0.85–2.52, *p* = 0.166). This indicates that the above described 30% provides an optimal cutoff point for TILs in TME‐based risk stratification. A 50% cutoff point was optimal for TSR in our TME‐based risk stratification.

## 4 Discussion

Tumor microenvironment has a significant role in cancer progression [18]. Recently, stromal‐related prognostic biomarkers have been introduced for risk assessment in head and neck cancers [9]. Identification of stromal markers can aid in targeting tumor‐associated stromal cells for cancer therapy [19]. In daily practice of OPSCC, however, TME is not considered during the management of oropharyngeal cancer. In addition, it is sometimes challenging to select the most suitable treatment for OPSCC patients [20]. In the present study we have introduced for the first time in a large cohort of OPSCC a TME‐based grading system that can be evaluated using routine hematoxylin and eosin (HE)‐stained slides and, therefore, can be easily included in pathology reports without additional costs. Our proposed system combines features of stromal microenvironment and immune microenvironment and has shown a powerful prognostic value in risk stratification of OPSCC.

Interactions of cancer cells with cells of tumor stroma are complex and implicated as key players in cancer invasion. During cancer progression, cancer cells and other components modify stromal cells to form a phenotype that promotes tumor development [19]. Tumor stroma can regulate tumor growth and it has the potential of regulating the aggressiveness of the tumor. Thus, research efforts on novel therapeutic strategies aim at targeting anti‐tumoral and anti‐stromal agents [21].

Research efforts which have included immune parameters as prognostic classifiers have shown promising findings [10]. Importantly, the assessment of stromal TILs is the most widely used immune parameter and has been reported as a powerful prognosticator in recent studies on various tumors [22, 23, 24, 25]. It is necessary to point out that the prognostic value of intra‐tumoral TILs was limited (*p* > 0.05), as also previously reported in oral cancer [23]. In the current study, the method used for the assessment of stromal TILs is standardized, and it has shown a promising prognostic value and good inter‐observer agreement [22, 23, 24, 25]. In addition, the method is cost‐effective as the assessment of TILs is made on HE‐stained sections which are already available as diagnostic samples. Similarly, the assessment of TSR is cost‐effective and has shown a significant prognostic value and good reproducibility [6, 7, 8, 9].

Of note, recent research has proposed stromal‐related characteristics for risk stratification of different tumors to supplement the currently used tumor‐related features in risk stratification of cancer. Interestingly, two recent studies have reported the prognostic significance of stroma‐based stratification in oral squamous cell carcinoma [11, 12]. Our current study corroborates the results of these studies. Importantly, when using diagnostic biopsy sections for the assessment of TSR, it is necessary to have a representative sample including sufficient amounts of both tumor and stromal compartments. In such samples, a good agreement on the TSR score in pre‐treatment biopsies and surgical specimens has been reported in head and neck cancers [26, 27]. Interestingly, there is evidence indicating that TSR and response to neoadjuvant chemoradiotherapy are correlated so that in esophageal cancer stroma‐low tumors show a better response to neoadjuvant chemoradiotherapy [28]. In agreement to that, in esophagogastric junction adenocarcinoma the assessment of TSR in preoperative biopsies has shown a predictive power for neoadjuvant therapy response [29]. Indeed, further studies are needed to compare TSR in tumor specimens before and after radiotherapy/chemo‐radiotherapy.

In high‐income countries, HPV‐positive OPSCC is one of the most rapidly increasing cancers [2]. In addition, it is well documented that HPV status is a valuable prognostic parameter in classifying OPSCC into risk groups. Therefore, in the present study we have included HPV status as a prognostic parameter together with our proposed TME‐based risk stratification in the multivariable analyses (Table 2), and both parameters showed a significant prognostic value indicating prognostic independence of each. This also indicates that our proposed TME‐based risk stratification can provide a risk stratification beyond the HPV status. This is an important observation to support optimal treatment planning based on multiple prognostic indicators.

The clinical decision‐making in OPSCC is sometimes challenging as cases that are usually considered as low risk (particularly the HPV+ tumors), may still present with poor outcome [2]. Thus, additional prognostic factors are needed to optimize risk stratification to personalize the treatment and avoid both under‐ and over‐treatment. The findings of our study indicate that category III tumors of the proposed TME‐based risk stratification carry a high risk of recurrence and mortality and thus require close follow‐up. More importantly, they might require more aggressive treatment even when diagnosed at an early stage. In conclusion, the proposed TME‐based risk stratification is cost‐effective and has a valuable prognostic power in identifying OPSCC cases at high risk of poor outcome. After validation studies, TME‐based risk stratification can be incorporated in the routine pathology reports, and it can be considered for treatment planning for OPSCC patients. It is a shortcoming of the present study that the patient cohort was limited to the period 2000–2009. Thus, further validation in a recent, preferably large multicenter cohort is desirable.

## Conflicts of Interest

The authors declare no conflicts of interest.

## Data Availability

The data that support the findings of this study are available from the corresponding author upon reasonable request.
